# Individualized Cognitive Functional Therapy Compared with Conservative Treatment for Patients with Chronic Neck Pain—Study Protocol for a Single Blind Pragmatic Randomized Controlled Trial

**DOI:** 10.3390/clinpract14040098

**Published:** 2024-06-21

**Authors:** George Ploutarchou, Vasileios Korakakis, Evi Lazoura, Christos Savva, Kyriakos Pavlou, Iacovos Christofi, Katerina Sotiriou, Chara Savvidou, Andreas Panteli, Christos Karagiannis, Ioannis Mamais

**Affiliations:** 1 Department of Health Science, European University of Cyprus, Nicosia 2404, Cyprus; e.lazoura@external.euc.ac.cy (E.L.); k.pavlou@external.euc.ac.cy (K.P.); ic222450@students.euc.ac.c (I.C.); katerina.g.sotiriou@gmail.com (K.S.); charasavvidou1@gmail.com (C.S.); andreaspant18@gmail.com (A.P.); c.karayiannis@euc.ac.cy (C.K.); i.mamais@euc.ac.cy (I.M.); 2 Department of Health Sciences, University of Nicosia, Nicosia 2417, Cyprus; vkorakakis@hotmail.com; 3 Department of Life and Health Science, Federick University, Limassol 3080, Cyprus; c.savva@frederick.ac.cy

**Keywords:** chronic pain, cognitive functional therapy, neck pain, physiotherapy, clinical practice

## Abstract

Chronic neck pain (CNP) is one of the most common musculoskeletal conditions, is considered the second leading cause of pain, and is among the leading causes of disability. Cognitive Functional Therapy (CFT) is a novel behavioral therapy for individualizing the management of spinal pain targeting the multidimensional aspect of musculoskeletal pain. This study outlines the protocol for an assessor-blind randomized controlled trial (RCT) designed to compare an individualized Cognitive Functional Therapy (CFT) intervention with usual care in terms of pain and disability. Aiming for a pragmatic intervention, the CFT group will receive 16 sessions based on patient’s condition characteristics, and clinical presentation and progression. The control group will receive 16 sessions of standardized usual care (electrotherapy, massage, posture exercise, and educations). Both groups will have the same intervention duration. Patients will be randomly allocated into groups and will be assessed at baseline, at the 8th session, at the 16th session, and 3 months after randomization. Primary outcomes will be pain, disability, cervical range of motion, and neck muscle isometric strength. To our knowledge, this study will be the first RCT to compare the clinical effectiveness of CFT compared to UC for adults with CNP. The study results will provide information about the use of CFT in clinical practice.

## 1. Introduction

Regarding musculoskeletal disorders, chronic neck pain (CNP) is considered not only one of the most common disorders but also the second leading cause of musculoskeletal pain and disability worldwide [1]. CNP affects more females than males (female/male ratio of 3:1) in a range between 50 and 60 years of age [2]. Research shows that 57% of patients who are suffering from neck pain will continue experiencing pain for longer than 6 months, of which 37% will experience disability and pain for longer than 12 months [3,4]. Risk factors such as a previous traumatic event, high work demands, low social or work support, a smoking history, female sex, and the fifth decade of life are associated with the onset of neck pain (NP) [3]. Moreover, the chronicity of NP seems to be linked with a variety of psychological and social factors (high workload, passive coping style, stress, anxiety, depression, catastrophizing, etc.) [5,6,7,8,9].

Cognitive Functional Therapy (CFT) is an integral part of the biopsychosocial approach used in spinal pain management [10]. CFT is based on a multidimensional clinical reasoning framework and has three unique goals. Firstly, CFT enhances the clinician’s ability to distinguish the condition’s modifiable components; secondly, it recognizes behavioral responses to the pain of the patients and evaluates them; and thirdly, it leads the way to a personalized rehabilitation program based on self-management [10]. “Lifestyle change”, “making sense of pain”, and “exposure with control” are the three indispensable components that are incorporated into the intervention and interplay with each other [10,11]. To illustrate, “lifestyle change” aims to promote personalized and systematic exercise, promote better nutrition, and improve sleep, leading to the adoption of a less sedentary lifestyle and managing fear, stress, and anxiety [6,7]. “Making sense of pain” is considered to be a reflective process based on the Pain Neuroscience Education (PNE) principles [12] while its purpose is to help patients controvert their previous beliefs and embrace a novel point of view of their pain experience. Finally, “functional integration” intends to allow individuals to progressively return to functional activities without pain escalation and related distress. This approach challenges their fear-avoidance beliefs and utilizes experiential learning in a process of behavioral change [6,7]. It can be argued that most contemporary conservative treatment approaches do not address the biopsychosocial component of the condition through a behavioral, educational, and self-engaging lens. Therefore, we believe it is essential to evaluate whether a CFT is more clinically effective than individualized conservative treatment (CT).

Despite the research-proven efficacy of CFT in chronic low back pain, there is limited research in CNP [12].

**Primary objectives**

**Figure 1:**
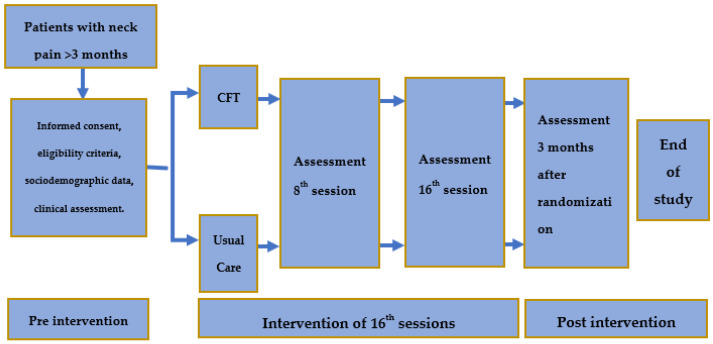
Schematic illustration of the study design. Abbreviations: CFT, Cognitive Functional Therapy.

The primary objective of this study is to examine the potential clinical effectiveness of an individualized CFT intervention compared to a usual care treatment in participants with CNP in pain (Numeric Pain Rating Scale—NPRS) and disability (Neck Disability Index) in the short- and mid-term follow up in a pragmatic assessor blind randomized controlled trial (RCT) with parallel groups (Figure 1).

**Secondary objectives**

The secondary objectives aim to evaluate fear perception, functionality, quality of life, range of motion, and strength in the short- and mid-term follow up.

**Trial design**

## 2. Materials and Methods

### 2.1. Methods: Participants, Interventions and Outcomes

#### 2.1.1. Study Setting

The study design is a two-armed RCT comparing individualized CFT and a usual care treatment in adults with CNP. The study procedures will be conducted from 15 August 2023 to 30 September 2024 (anticipated) at the European University of Cyprus, Nicosia, involving patients with received referrals from healthcare providers (we will inform them by email) or social media advertising. Any major modification that impacts the study will require a formal amendment to the protocol by the project supervisors (CK and IM) and approval by the ethics committee. The research has been fully approved and registered by the Cyprus Bioethics Committee (ΕΕΒΚ/ΕΠ/2023/20) (SupportingInfo 1–4).

To conduct and report this RCT, we will use the SPIRIT checklist and the CONSORT statement (SupportingInfo 9–10).

#### 2.1.2. Eligibility Criteria

Participants willing to participate in the study will be informed about the purpose of the study, its duration and details, and why it is important to participate in the study. Specifically, they will be given the information sheet with the relevant information (SupportingInfo 7–8). Participants will be screened individually for eligibility criteria. Eligible patients will be informed that there are two active interventions, and it is not known which one is superior. Participants will also receive a written consent form to sign (SupportingInfo 5–6). The inclusion/exclusion criteria are listed in Table 1. Interventions will be performed by two physiotherapists and two assistants (one main treating physiotherapist and one assistant per clinic) with more than 5 years of experience and formal education in CFT intervention [13,14].

#### 2.1.3. Interventions

All interventions will be performed by four physiotherapists (GP and EL) of which two will be assisting (KS and CS). Only one investigator (and one assistant) will be involved in the CFT arm of the trial to ensure reliability. The main investigator (GP) has 10 years of experience in cognitive and behavioral treatments and 2 years of clinical experience in CFT (has participated in a 3-day course—total 24 h), and the assistant in the CFT group has 1 year of experience in physiotherapy practice. The second investigator with more than 5 years of experience in physiotherapy practice (EL) will perform all interventions in the CT group, with one physiotherapist as an assistant (CS) with 1 year of clinical experience. For both intervention groups, all physiotherapists will pilot the interventions until they are deemed confident for deliverance. Assistants will aid in documentation and management.

##### Individualized CFT

CFT intervention will be individualized and will involve the patient’s subjective, objective, and physical examination by the treating physiotherapist. During the interview, participants will provide data regarding their medical history, pain distribution, pain during the day, movement/s aggravating or easing symptoms, functional impairments, disability levels, medication, quality of life, perception, and beliefs about pain. Also, at the interview, participants will describe their psychological perception of their ongoing health problems such as pain focus degrees, fear of pain, avoidance activities, and work and social engagement. Finally, their goals will be written and discussed. Based on the findings of the subjective assessment, the treating physiotherapist, via discussion with the patient, will prioritize the components of the CFT approach according to the needs, beliefs, and perceptions of the patient. The physical examination will include a full neurological examination, cervical range of motion assessment, and isometric strength measurement for flexion, side flexion, rotations, and extension of the cervical spine.

Treatment will be provided at the university’s lab, and the initial session will last approximately 90 min, while the follow up sessions will range from 45 min to 1 h. The frequency of the treatment will be twice a week; however, it will be accordingly reduced in frequency over time, and the total number of sessions will be 16. The intervention will contain the following but individualized components:

Cognitive components: education about patient’s perceptions of pain, diagnosis explained, diagnostic finding analysis, answer any question about their problem, symptoms, or anything else that comes up. We will progressively challenge their beliefs in a non-judgmental way and educate them about the multifactorial and biopsychosocial spectrum of pain. All participants will be encouraged to move, stay active, and participate in daily activities. Additionally, patients will receive a self-management plan, which includes stretching, breathing exercises, postural relaxation techniques, self-massage, participation in activities with 2–3/10 difficulty, and sleep hygiene advice if needed.

Specific functional training: This will involve body awareness and control exercises and body relaxation exercises in order to relax and start to understand how to modify the pain. Additionally, participants will be taught modified postures to control their cervical area whilst relaxing their thoracic spine, enabling them to engage in activities that are painful or fearful. Also, at this point the participants will engage in daily activity movements like sitting and rolling in a chair, sitting, standing up from sitting, walking, bending, and lifting.

Lifestyle changes: The promotion of gradually increasing physical activity based on their preference, stress management, and social interaction.

##### Conventional Therapy

Treatment (one-to-one) will be provided at the university’s lab; the initial session will last approximately 90 min, and follow up sessions will range from 45 min to 1 h. The frequency of the treatment will be twice a week; however, it will be accordingly reduced in frequency over time, and the number of sessions will be approximately 16 sessions. The intervention will contain the following components:

Transcutaneous electrical nerve stimulation (TENS) (Frequency <200 Hz, pulse duration <250 μs for 20 min), massage (cervical area for 15 min), isometric exercise using patients’ hands, for flexion, extension, side flexion, and rotations of the cervical spine (all 3 sets of 10 repetitions, with resistance directed by the patient according to their symptoms, endurance, and capacity), chin-in exercise (3 sets by 10 repetitions, in a slow pace and a 2 s hold), and education about sitting or lifting posture (contemporary ergonomic advice and information of interrupting prolonged postures).

#### 2.1.4. Outcomes

Primary outcomes will be self-reported except for isometric strength and cervical range of motion, and will be conducted at baseline, 8th session (week 4), and 16th session (week 8), as well as at 12 weeks after the randomization. The primary outcome will be the NPRS—a valid 0–10 scale with a 1.5-point change as a minimum clinically important difference (MCID)—and the Neck Disability Index—a questionnaire consisting of 10 questions rated in a 6-point Likert scale with 0 indicating no disability and 5 indicating major disability with an MCID of 25% [15].

Secondary outcomes will be the perception of fear using the four-item physical activity subscale of the Fear Avoidance Beliefs Questionnaire, functionality using Short Form 12 (SF12), and the EuroQuol-5D for quality of life [8,16,17].

All questionnaires are translated and cross-culturally adapted for Greek-speaking individuals and have been previously used in studies with patients suffering from neck pain [18,19,20,21].

#### 2.1.5. Participant Outcome Assessment Timeline

Self-reported questionnaires will be administered and completed at baseline and at follow up at the end of the 8th session (week 4), at the end of the 16th session (week 8), and at 12 weeks after randomization. The outcome assessor (AP) will be blinded to the intervention allocation, and participants will be partially blinded as they will be informed that two active therapies will be applied but will not be given information about the alternative treatment. At the end of the intervention, they will fill out a questionnaire that will ask if they can identify which group they were in.

#### 2.1.6. Sample Size

Based on the available evidence using CFT for CNP, a sample calculation suggested a sample size of 26 participants per group. We will add an additional 15% in the case of dropouts, so the final sample size will be set at 30 participants per group, 60 in total. The effect size for pain reduction in an NPRS was set at 0.80, the α error probability at 0.05, and the power (1-β) at 0.80 using the G*POWER 3.1 calculation program [12].

#### 2.1.7. Recruitment

We will use convenience sampling to recruit the participants via social media posts and/or through referrals by healthcare providers (orthopedists, physiotherapists, and general practitioners). We will prepare an advertising post for social media with study criteria and contact information. We will send emails with the information and criteria to two orthopedic doctors, two physiotherapists, and two general practitioners; in case a patient is matched to our research, they will be able to contact the main researcher (GP) and arrange an assessment appointment via telephone or email.

### 2.2. Methods: Assignment of Intervention

#### Treatment Allocation, Randomization, and Blinding

We will use a block randomization process (block size of 6). A piece of paper from an envelope will be picked by every block’s first patient, letter A will be for CFT and letter B for CT. The rest block allocation is indicated by the first-person choice. The selection will be concealed in sequentially numbered, sealed, opaque envelopes, and kept by an independent assistant (AP), who will be responsible for the group allocation. Participation in data collection and treatment procedures will be minimal. The assistant will be blinded to group allocation and the block size. Similarly blinded to group allocation will be the statistician who will analyze the final data [22].

### 2.3. Methods: Data Safety Monitoring, Treatment Fidelity, and Statistical Analysis

#### 2.3.1. Data Safety Monitoring

For responsibilities such as data collection from the assessor, data transfer, and data analysis, the person in charge will be the main investigator. During the treatment sessions, the participants’ health will be monitored. Local health service providers will be contacted by the supervisors (CK and IM) in the case of adverse events and in the improbable situation where harm is suffered. In the final written report of this study, every improbable event will be included. All study data will be stored securely at the European University of Cyprus. All paper-based documents and data will be stored in a secure locker. The security of all electronic data will be achieved with the use of a password-protected laptop.

#### 2.3.2. Data and Treatment Fidelity

A record with the type and number of treatments will be held for every patient in the study. For both groups, the treating physiotherapist will document a session-by-session treatment content which then will be double-checked by the assistant. Additionally, a week-by-week meeting will be scheduled by both the main treating physiotherapist and assistant in order to discuss the procedures, dropouts, and any other problems that arise. The main physiotherapist will train the assessor in the history-taking process, the physical examination, and the administration of the questionnaires.

#### 2.3.3. Statistical Analysis

Descriptive statistics will be used to summarize the baseline characteristics of the participants and the measurements. The residuals of each variable/model will be checked for normality by visual inspection of the frequency histograms and the Q-Q plots and using the Shapiro–Wilk test.

Mixed-effects models will be used for data analysis, and for each outcome of interest, the best model will be used based on the Akaike information criterion. The analysis of the outcome variables (NPRS, NDI, FABQ, SF-12, EQ-5D, ROM, and isometric cervical spine strength) will be carried out using subject-specific random effects over the three assessment time points (baseline, 8 sessions, 16 sessions, and 12 weeks). Group allocation (CFT or CT), the time of outcome assessment, and all interactions will be included in the fixed-effects model. We will model the parameter estimates for baseline covariates associated with recovery (age, weight, height, body mass index (BMI), etc.)

Where a significant main effect or interaction is found, we will perform post hoc testing and Tukey adjustment for multiple comparisons. All data will be analyzed using JMP (v.16.0, SAS), and the level of significance will be set at 0.05.

#### 2.3.4. Ethics and Dissemination

The relevant Cyprus National Bioethics Committee (ΕΕΒΚ/ΕΠ/2023/20) granted our research with ethical approval. All participants included in the study will receive a written information letter. Participants will be aware of their right and ability to withdraw from the study at any time without having any negative consequences on their future care. The privacy protection of the participants will be guaranteed, as any type of personal information (name, etc.) will always be confidential. Events like soreness and stiffness are expected in both groups because of exercise, but other than that, no significant adverse event is expected. Nevertheless, with the aim of reducing stiffness perception, participants will perform the exercises at their own speed and intensity, but also, after the exercise, a smooth massage will be performed.

## 3. Discussion

CFT effectiveness has been mainly evaluated in LBP patients [7,13,23,24]. A recent systematic review assessed the effectiveness of CFT in LBP patients [7] and presented very low certainty favorable outcomes of the intervention on pain intensity (long-term), disability, and fear of movement. Unfortunately, the generalizability of these results is limited due to the heterogeneity of the comparators and the small number of included studies [7].

In the present RCT, we aimed to compare CFT with usual care; however, there is only one clinical trial available with a similar comparison [12]. Nevertheless, the study that compared CFT with a control group has significant limitations, and we will try to improve the current literature using a more robust methodology in terms of study design, sample size, follow up, and outcome measures as illustrated in Table 2 [12].

The strength of our study is mainly based on the pragmatic approach to CFT intervention. The available literature uses CFT as a standardized non-individualized protocol that is provided to patients with CNP [12]. In the present study, we aim for an individualized intervention that will offer pragmatic and useful data directly informing clinical practice. We acknowledge that such an intervention is not reliably replicable; however, physiotherapy practice is informed by general clinical guidelines that are filtered through patient needs and symptoms using clinical reasoning. Thus, this will be the first RCT to compare the clinical effectiveness of an individualized CFT intervention to usual care for adults with CNP.

## 4. Limitations

We acknowledge that the main limitation of the present study is the lack of blinding to the personnel who provide treatments; however, given the nature of the intervention, this was not possible.

## 5. Conclusions and Clinical Implication

This will be the first RCT to compare the clinical effectiveness of individualized CFT compared to usual care for adults with CNP. The study results will provide information about the use of CFT in clinical practice with the aim of informing and potentially improving the management of CNP.

## Figures and Tables

**Table 1:** Inclusion and exclusion criteria for study participation.

Inclusion Criteria	Exclusion Criteria
Aged between 18 and 64 years	Pain less than 3 months
Chronic neck pain for at least 3 months	Recent neck or shoulder surgery (<6 months)
Score of 2 points or more on Numeric Pain Rating Scale (NPRS) from 0–10	Progressive neurological disease (e.g., multiple sclerosis, Parkinson’s, motor neuron disease),
Be independent to arrive at the location and participate in the rehabilitation program	Red flags like cancer, fracture (<6 months) or infection, cauda equina, spinal cord compression
Greek-speaking	Rheumatological/inflammatory disease (e.g., rheumatoid arthritis, ankylosing spondylitis, psoriatic arthritis, Scheuermann disease)
	Spinal stenosis
	Pain relieving invasive procedures such as injection-based therapy in the past 3 months
	Unstable cardiac conditions
	Serious psychopathic pathology

**Table 2:** Main difference between the previous and present study.

	Javdaneh et al.’s Study [12]	Present Study
Type	Clinical trial	Randomized controlled trial
Participants	24 (24–40 years) (only men)	60 (18–64) (both sexes)
Intervention	Usual care not described	Fully described
Duration of intervention	6 weeks—18 sessions	8 weeks—16 sessions
Follow up	No follow up	3 months from the randomization
Outcomes	Self-efficacy, pain catastrophizing, fear avoidance, anxiety, depression, stress	Pain, disability, kinesiophobia, quality of life, functionality, isometric strength, range of motion

## Data Availability

No data available yet.
